# Greater trochanter morphology and association with patient demographics, surgical factors, and post-operative stem position: a retrospective assessment of 150 cementless THRs in 135 dogs

**DOI:** 10.1186/s12917-022-03174-y

**Published:** 2022-02-23

**Authors:** Catrina J. Silveira, Katherine H. Barnes, Sharon C. Kerwin, W. Brian Saunders

**Affiliations:** Department of Small Animal Clinical Sciences, College of Veterinary Medicine & Biomedical Sciences, Texas A&M University, 4474 TAMU, College Station, TX 77843-4474 USA

**Keywords:** Total hip replacement, THR, Greater trochanter, Classification, Cementless THR

## Abstract

**Background:**

Total hip replacement (THR) in the gold standard surgical treatment for the canine hip. While it has been shown that greater trochanter morphology affects post-operative cementless stem position in humans, trochanter morphology and the effect on cementless stem position has not been extensively evaluated in dogs. The objective of this study was to classify greater trochanter morphology and identify potential associations between trochanter morphology and patient demographics, femoral canal geometry, surgical time, technique modifications, and post-operative stem position in client-owned dogs undergoing cementless THR.

**Results:**

In this retrospective study, medical records and radiographs of 135 dogs undergoing 150 cementless total hip replacements from 2013 to 2020 were included. Trochanters were classified in the frontal plane using an ordinal grading system adapted from human THR. A Grade I trochanter denoted a trochanter positioned lateral to the periosteal surface of the lateral femoral cortex, whereas a Grade IV trochanter denoted a trochanter positioned medial to the anatomic axis of the femur. Associations between trochanter grade and other variables were examined using ANOVA, Kruskall-Wallis, or chi-squared tests. Significance was assumed at *P* ≤ .05.

Trochanters were classified as follows: Grade I (44/150, 29.3%), Grade II (56/150, 37.4%), Grade III (44/150, 29.3%), Grade IV (6/150, 4.0%). Grade IV trochanters had lower anatomic lateral distal femoral angle (aLDFA; 91.0 ± 6.2°), angle of inclination (117.7 ± 10.5°), and canal flare index (1.53 ± 0.27). When compared to all groups, Grade IV trochanters were associated with longer surgical times (Grade IV: 227.0 ± 34.2 min; all grades: 183.2 ± 32.9 min) and technique modifications (Grade IV: 83.3%; all grades: 18%). Grade I trochanters had stems placed in valgus (− 1.8 ± 2.33°), whereas Grade II (0.52 ± 2.36°), III (0.77 ± 2.58°), and IV (0.67 ± 2.73°) trochanters exhibited varus stems. Depth of stem insertion was greater (11.2 ± 4.2 mm) for Grade IV trochanters.

**Conclusions:**

Trochanter grade was associated with post-operative stem alignment and translation in the frontal plane. Grade IV trochanters were associated with altered femoral geometry, increased surgical time, technique modifications, and stem insertion depth. Pre-operative greater trochanter classification may prove useful in identifying cases requiring prolonged surgical times or technique modifications.

**Supplementary Information:**

The online version contains supplementary material available at 10.1186/s12917-022-03174-y.

## Background

Common indications for canine total hip replacement (THR) include hip dysplasia, traumatic hip luxation, femoral head, neck, or acetabular fractures, and avascular necrosis of the femoral head [1–4]. Although complications can occur, successful clinical outcomes have been reported in 83–95% of cases [5–11]. Historically, canine THR was performed using cemented implant systems [1, 5]. Presently, the majority of canine THRs are performed using cementless implant systems [6, 12–14].

Proper femoral canal preparation is essential for successful press-fit cementless THR [15–17]. Key steps include identification of the anatomic axis of the femur in both frontal and sagittal planes, centralized canal entry, insertion of sequential reamers and broaches to create a press-fit envelope, followed by stem insertion and impaction [4, 18]. Complications associated with improper broaching or stem malalignment include stem subsidence, femur fissure or fracture, failure of osseointegration, and prosthesis luxation [17, 19–23]. In human THR, several factors have been associated with post-operative cementless stem position. These include surgical approach, limb positioning and retraction, broaching technique, presence of proximal femoral sclerosis, and abnormal femoral anatomy [24–28].

The shape and location of the greater trochanter affects femoral canal entry, broaching, and subsequent stem position. In the frontal plane, a markedly medialized trochanter (often referred to as a medially overhanging trochanter) creates specific challenges during THR. These include reduced exposure and access to the femoral canal, iatrogenic damage to the gluteal tendons, increased risk of intra-operative femur fracture, and post-operative stem malalignment [17, 29–31]. Techniques to objectively assess and classify trochanter position relative to the trochanteric fossa and anatomic axis of the femur have been described in human THR patients [22, 29]. These classification techniques are recommended pre-operatively during THR templating to guide intra-operative broaching and stem placement.

Greater trochanter osteotomy (GTO) has been used to address the medially overhanging trochanter, improve access to the trochanteric fossa for canal entry, preserve gluteal musculature, and improve broaching accuracy [32–34]. In veterinary orthopedics, GTO is most commonly associated with fixation of acetabular fractures [35]. GTO has also been described as a method to improve exposure in primary or revision THR as well as to facilitate surgical management of chronic hip luxations [36, 37]. A recent case series described the pre-operative planning and surgical technique for GTO and cementless THR in dogs with the luxoid phenotype of hip dysplasia or chronic craniodorsal hip luxations [38].

Although subjective assessment of trochanter position is recognized as an important component for canine THR templating [4, 18], techniques to objectively define trochanter position in dogs undergoing THR have received little attention. Using computed tomography (CT) as the gold-standard, Davis and colleagues validated radiography as a method to assess trochanter position and described the incidence of medially positioned greater trochanters in a population of dogs with and without hip dysplasia [39]. However, the association between trochanter position, surgical factors, or post-operative cementless stem position were not a focus of the study. In another study, Alvarez-Sanchez and colleagues described the pre-operative radiographic abnormalities and outcomes in dogs with capital physeal fractures treated with a cementless THR system. The position of the greater trochanter was radiographically assessed for medial overhang relative to the central axis of the medullary canal on oblique lateral radiographs in 25° of supination, which is unique radiographic projection to the cementless THR system used in that study [40]. Therefore, the objectives of the present study were to classify frontal plane greater trochanter morphology using traditional radiographic views in a cohort of dogs undergoing cementless THR and to identify potential associations between trochanter morphology and patient demographics, femoral geometry, surgical time, technique modifications, and post-operative stem position.

## Results

### Demographic variables

In this exploratory study, a total of 150 THRs were included for evaluation. Fifteen dogs received bilateral THRs. As such, there were 135 individual dogs. The most common breeds included Labrador Retriever (28), German Shepherd Dog (14), Golden Retriever (11), Pit Bull Terrier (7), Great Pyrenees (6), Australian Shepherd (5), Border Collie (5), Rottweiler (5), Australian Cattle Dog (4), Cane Corso Mastiff (4), Husky (3), and Saint Bernard (3). A complete breed list is provided in Supplemental Table 1. There were 52 (39%) castrated males, 12 (9%) intact males, 67 (50%) spayed females, and 4 (3%) intact females. Age, body weight, and underlying diagnoses are provided in Table 1. Greater trochanter grade was not associated with patient age (Kruskal-Wallis, *P* = 0.93), body weight (ANOVA, *P* = 0.39), or underlying diagnosis leading to THR (χ^2^ = 7.03; *P* = 0.63).

**Table 1:** Demographic data based on greater trochanter grade

	I		II		III		IV		All	
	Mean	SD	Mean	SD	Mean	SD	Mean	SD	Mean	SD
Age (months)	34.4	27.8	34.4	31.1	35.6	28.3	37.7	36.0	34.9	29.2
Body weight (kg)	30.0	8.5	33.2	10.4	32.0	9.0	30.0	4.7	31.8	9.3
Diagnosis	Number	%	Number	%	Number	%	Number	%	Number	%
Hip dysplasia (OA)	20	45.5	31	55.4	23	52.3	2	33.3	76	50.7
Luxoid hip	16	36.3	14	25.0	16	36.4	2	33.3	48	32.0
Traumatic hip luxation	4	9.1	9	16.1	3	6.8	1	16.7	17	11.3
Fracture	4	9.1	2	3.5	2	4.5	1	16.7	9	6.0
Total numbers	44	100.0	56	100.0	44	100.0	6	100.0	150	100.0

### Pre-operative radiographic findings

Trochanter morphology was classified as follows: Grade I (44/150, 29.3%), Grade II (56/150, 37.4%), Grade III (44/150, 29.3%), Grade IV (6/150, 4.0%) (Table 1). Additional pre-operative femoral measurements are provided in Table 2. There were no associations between trochanter grade and femoral torsion angle (FTA) or cortical thickness ratio (CTR). There were associations between trochanter grade and the anatomic lateral distal femoral angle (aLDFA), angle of inclination, and canal flare index (CFI).

**Table 2:** Pre-operative radiographic measurements of femoral geometry based on trochanter grade

	I		II		III		IV		All		*P* value
	Mean	SD	Mean	SD	Mean	SD	Mean	SD	Mean	SD	
aLDFA (°)	94.1	3.5	95.1	3.4	95.4	4.6	91.0	6.2	94.7	4.0	0.04*
Inclination (°)	128.3	8.2	127.4	5.9	125.6	8.5	117.7	10.5	126.7	7.8	0.01*
FTA (°)	39.1	12.4	38.4	8.8	39.3	9.6	34.3	8.0	38.7	10.1	0.71
CFI (ratio)	1.96	0.27	1.81	0.21	1.73	0.19	1.53	0.27	1.79	0.24	0.002*
1/CTR	2.84	0.48	2.93	0.74	3.08	0.66	3.51	0.67	2.97	0.66	0.07^

*Key*: *aLDFA* anatomic lateral distal femoral angle, *CFI* canal flare index, *FTA* femoral torsion angle, *1/CTR* inverse of cortical thickness ratio

*: significant difference found in ANOVA, then followed by Scheffe’s post hoc test. *aLDFA*: no significant differences between any specific pairs. *Inclination*: Grades I and II different from Grade IV. *CFI*: Grade I and II different from grade IV

^Inverse CTR (inverted because of data distribution before transformation)

### Surgical factors

All THR were performed by a single surgeon with 14 years of primary THR experience (WBS). Dogs received Ti-EBM BFX® femoral stems as follows: BFX™-lateral bolt (*n* = 93), BFX™-standard (*n* = 32), and BFX™-collared® (*n* = 25). Selection of BFX™ stem was based on surgeon preference and implant availability at the time of surgery. The most common BFX stem size was 8 mm (range 5–12). Mean surgical time was 183 ± 33 min. Grade IV cases had prolonged surgical times as compared to other trochanter grades (Table 3). Technique modifications were recorded in 27/150 THRs (18%) and disproportionately involved Grade III and IV cases, with four THRs noted to involve two technique modifications (Supplemental Figure 1). Modifications included use of aggressive-toothed femoral files (instrumentation circa 2005, Biomedtrix, Whippany, NJ, USA) to deplete medialized trochanter and/or sclerotic bone in order to achieve proper broach alignment [17], GTO for improved canal access during broaching in Grade III or IV cases [8], prophylactic cerclage placement in sclerotic femurs [4], use of a Hall® pneumatic high-speed burr to deplete the medial aspect of the trochanter and sclerotic bone to achieve proper broach alignment [1], and intentional downsizing of a cementless stem to reduce neck length in a dog with femoral sclerosis and chronic craniodorsal hip luxation [1]. There were no intraoperative complications.

**Table 3:** Surgical factors and frontal plane post-operative stem position values based on trochanter grade

	I		II		III		IV		All		*p* value
	Mean	SD	Mean	SD	Mean	SD	Mean	SD	Mean	SD	
Surgical time (min)	177.5	32.8	179.8	27.9	187.5	34.7	227.0	34.2	183.2	32.9	0.003*
Technique mod (% of cases/group)	11.4		10.7		25.0		83.3		18.0		< 0.001*
Stem alignment in frontal plane (°)	−1.80	2.33	0.52	2.36	0.77	2.58	0.67	2.73	−0.47	2.61	< 0.001*
Stem translation in frontal plane (ratio)	−0.07	−0.09/0	− 0.07	−0.08/0	0	−0.07/0	− 0.09	−0.08/0	−0.07	−0.08/0	0.012^
PO-FTA (°)	18.8	14.0	20.4	14.4	20.8	9.2	20.6	5.5	20.0	12.6	0.93
Canal fill in frontal plane (%)	73.0	6.3	73.3	6.9	71.1	7.1	66.2	10.0	72.3	7.0	0.06
Stem insertion (mm)	7.0	2.7	6.1	3.0	7.0	2.9	11.2	4.2	6.8	3.1	0.001*

*Key*: *PO-FTA* post-operative femoral torsion angle, *Stem depth* post-operative stem insertion depth relative to the proximal greater trochanter, *Technique mod* technique modification during femoral broaching

*: significant difference found in ANOVA, then followed by Scheffé’s post hoc test. *Stem alignment*: Grade I differs from Grade II. *Surgical time*: Grade IV different from all other groups. *Stem insertion*: Grade IV different from all other groups. *Technique mod*: Grade IV different from all other groups

^: Non-normally distributed data reported as median and interquartile range. Kruskall-Wallis test with Dunn’s post hoc test: Grade I and Grade III are different

### Post-operative radiographic evaluation

Measurements of frontal plane post-operative stem position are provided in Table 3. There were no significant associations between trochanter grade and post-operative frontal plane canal fill or post-operative stem anteversion as assessed by FTA. There was an association between trochanter grade and stem alignment in the frontal plane, stem translation in the frontal plane, and depth of stem insertion (Table 3). In Grade I cases, stems were placed in slight valgus, whereas in Grades II, III, and IV cases, stems tended to be placed in slight varus. Cementless stem insertion depth relative to the proximal most aspect of the greater trochanter was significantly greater in Grade IV cases.

There were no associations between trochanter grade and sagittal plane stem alignment or sagittal plane stem translation (Table 4). There was an association between trochanter grade and sagittal plane canal fill, due to lower canal fill for Grade IV cases.

**Table 4:** Sagittal plane post-operative stem position values based on trochanter grade

	I		II		III		IV		All		*p* value
	Mean	SD	Mean	SD	Mean	SD	Mean	SD	Mean	SD	
Canal fill in sagittal plane (%)	60.9	8.5	59.6	8.9	59.3	10.5	48.2	10.4	59.5	9.5	0.023*
Stem alignment in sagittal plane (°)	−0.07	2.48	0.71	2.23	0.82	2.66	0	2.53	0.49	2.45	0.289
Stem translation in sagittal plane (ratio)	0.03	0.06	0.04	0.06	0.03	0.07	0.06	0.07	0.04	0.06	0.557

*Key*: *: significant difference found in ANOVA, then followed by Scheffé’s post hoc test. *Canal Fill*: Grade IV different from Grade I and Grade II

## Discussion

The objectives of this study were twofold. The first was to describe a technique to classify greater trochanter morphology in the frontal plane in canine cementless THR patients and to report the incidence of trochanter grades in a cohort of dogs undergoing cementless THR. The described technique was practical, broadly applicable across numerous breeds of dogs, accessible with basic radiographic viewing software, and was based on previous work in the human THR literature [29]. Using a similar but not identical methodology in a population of normal and dysplastic hips, Davis et al. reported that 41% of greater trochanters were positioned medial to the lateral endosteal cortex, which are represented by both Grade III and IV trochanters in the present study. Collectively, Grade III and IV represent 33.3% of trochanters in the present study, which is similar to the 41% reported by Davis and colleagues. Davis quantitatively assessed the displacement of the trochanter medial or lateral to the lateral endosteal cortex of the femur, whereas the present study utilized an ordinal scoring system originally described by Wang (with slight modifications) [29]. One potential advantage of the scoring system described in the present study is the ability to quickly identify the more severely lateralized (Grade I, 29.3% of cases) or medialized (Grade IV, 4% of cases) trochanters. As these trochanter grades were associated with valgus or varus stem positions (Table 3), the ordinal system utilized herein may prove useful in identifying these cases during pre-operative THR planning. Additionally, Grade IV trochanters were associated with an increased incidence of technique modifications and prolong surgical times, which is an important association for perioperative surgical decision making and case scheduling.

The second objective of this study was, in an exploratory nature, to identify associations between trochanter grade and various clinical parameters including patient demographics, common radiographic measurements of femoral geometry, surgical time, use of technique modifications during THR, and post-operative stem position. There were no associations detected between trochanter grade and age, breed, sex, body weight, or underlying diagnosis leading to THR. The lack of association of trochanter grade and underlying diagnosis was noteworthy. A recent study described a high incidence of medialization of the trochanter in dogs with chronic capital physeal fractures (range: 45–2920 days) treated with a non-press fit cementless THR system [40]; however, that study contained some cases with fractures of several years duration prior to THR, and cases were not evaluated in the context of other pathologies of the hip as was performed in the present study. Additionally, the study by Alvarez-Sanchez and colleagues assessed trochanter position using a THR system-specific “25° of supination” lateral femoral radiograph, whereas in the present study trochanter grade was determined from traditional craniocaudal radiographs. Lastly, Alvarez-Sanchez and colleauges classified trochanters categorially in one of two groups, whereas in the present study an ordinal, four-group grading system was used. As such, it is difficult to make direct comparisons between the two studies.

Body condition score was not consistently present in medical records and, as such, was not evaluated. Interestingly, there were associations between common radiographic measurements and trochanter grade, including canal flare (CFI), angle of inclination, and aLDFA. These differences were primarily due to grade IV cases, which exhibited lower CFI, angle of inclination, and aLDFA values (Table 2). These findings support the authors’ clinical experience with severely medialized greater trochanter cases, which are often accompanied by lower inclination angles and slight lateral deviation of the diaphysis of the femur, resulting in reduced aLDFA values. There were not associations between trochanter grade and the pre-operative femoral torsion angle (FTA). This was likely due to the fact that trochanter grade is closely linked to radiographic measurements in the frontal plane (craniocaudal view), whereas the FTA is quantitatively determined using both frontal and sagittal plane images [41, 42].

In regards to surgical factors, there was a strong association between trochanter grade and surgical time and the number of recorded technique modifications during THR. Grade IV trochanters were associated with longer surgical times (Grade IV: 227.0 ± 34.2 min; all cases: 183.2 ± 32.9 min) and technique modifications (Grade IV: 83.3%; all cases: 18%). Three of the six Grade IV cases received greater trochanter osteotomies at the time of cementless THR, and the remaining cases were treated with aggressive-toothed files or a Hall® pneumatic high-speed burr to facilitate proper canal access, femoral broaching, and stem placement (Supplemental Figure 1). The high incidence of technique modifications, particularly GTO and subsequent pin and tension band fixation, is the likely explanation for the prolonged surgical times reported for Grade IV cases. It is important to note that a single surgeon with 14 years of THR experience performed all the THRs in the present study. While the technique modifications described above were used to address Grade III and IV trochanter cases in the present study, other experienced THR surgeons may have not utilized these specific modifications, or may have preferred other modifications not used in the present study.

Regarding stem position, there were associations between trochanter grade and frontal plane stem alignment, stem translation, and depth of stem insertion. In human joint replacement and traumatology, trochanter shape affects both cementless THR stem position and interlocking nail position [29, 43]. While it is well known amongst veterinary THR surgeons that femoral morphology plays an important role in surgical decision making [4, 18], the present study is the first to link trochanter classification objectively to post-operative cementless stem position. As expected, Grade I trochanters were associated with stems placed in slight valgus, whereas grade II – IV trochanters were placed in slight varus (Table 3). This association likely exists due to the support that the medial aspect of the trochanter provides to the lateral aspect of the femoral broach during canal preparation. A laterally positioned trochanter (Grade I) places the contact point between the medial trochanter and the lateral broach surface in a lateral position relative to the anatomic axis of the femur. The surgeon’s tendency in this setting is to insert the femoral broaches and stem in slight valgus, due to either resting the broach against the lateralized trochanter, or due to the fact that the cortical bone of the femoral neck on the medial aspect of the femoral canal preparation displaces the broaches laterally in the absence of trochanter support. In contrast, a medialized trochanter moves the contact point between the trochanter and broach medially. The surgeon’s tendency is to insert the broaches and stem in varus. These findings are consistent with a previous study assessing the effect of trochanter morphology on stem position in human beings undergoing cementless THR [29]. While there were associations between trochanter grade and stem alignment, these differences were admittedly small. This may be explained by the experience level of the surgeon, the ability of the surgical team to subjectively recognize trochanter morphologies during pre-operative templating, and the ability to adapt to patient-specific femoral anatomy intra-operatively during canal preparation and stem placement.

There was also an association between trochanter grade and frontal plan stem translation. Of all of the data sets in the present study, this was the most problematic to analyze. Stem translation measurements were performed with an accepted technique [8, 20] using a ratio that adjusts for individual dog size. This resulted in a non-normally distributed data set that was not amenable to data transformations. This explains why this data set was reported as median and interquartile range and analyzed using non-parametric analytics. The data indicate that Grade I trochanters were associated with stems that were translated in the lateral direction, whereas Grade III trochanters are associated with stems that are coaxially placed (Table 3). These findings are plausible, as a lateralized trochanter would allow the long axis of the broach to drift lateral to the anatomic axis of the femur. Of interest, however, is the finding that Grade IV cases had stems placed in a lateralized position. A reasonable explanation for this specific finding is related to the high incidence of GTO in Grade IV cases. Upon removal of the greater trochanter, the support and resistance provided to the broach is removed, thus allowing the broaching axis to fully translate laterally into contact with the lateral endosteal surface of the femur. Due to the distribution of these data, the authors caution readers not to over interpret these findings. In future study, additional techniques may be warranted to quantify stem translation in the frontal plane and generate normally distributed data sets.

Lastly, there was an association with trochanter grade and stem insertion depth and canal fill in the sagittal plane (Tables 3, 4). Canal fill in the frontal plane for Grade IV trochanters were lower than other grades (Table 3), but this difference did not reach significance. As mentioned above, several of the Grade IV trochanter cases were treated with GTO in conjunction with THR. In this setting, it is necessary to place the cementless stem distally, below the level of the osteotomy in order to reduce and stabilize the greater trochanter [38]. This is the likely explanation for the stem insertion depth results. In regards to CFI, Grade IV trochanters were associated with lower CFI. It has previously been shown that CFI and cementless stem canal fill are closely related [8]. Importantly, the cementless THRs in the present case series were second generation electron beam melted titanium stems. This generation of stems exhibits a reduced width in the distal 1/3 of the stem as compared to first generation cobalt-chrome stems. As canal fill is classically measured from distal to proximal [8, 20], the reduced canal fill reported in the present study is predicted when compared to work involving cobalt-chrome cementless stems. Additionally, a large proportion of cases were treated with either BFX™-lateral bolt (93/150) or BFX™-collared® (32/150) stems, which involve ancillary fixation and have previously been reported to exhibit reduced canal fill [23].

### Limitations

As with all studies, this study is not without limitations. While the methods described in the present study to classify trochanter grade pre-operatively are broadly applicable to all canine THR surgeons, the association of trochanter grade with surgical time, technique modifications, and stem position may not translate to other canine non-press fit, cementless systems THR systems. The cementless THR system utilized in the present study relies on a press-fit technique of a femoral stem placed within the medullary canal and is associated with a high degree of canal fill. Other commercially available canine cementless systems are not press-fit. These systems are designed to either occupy the medullary canal, relying on screw fixation for initial stability, with subsequent bone ongrowth or ingrowth [6, 44]. Alternatively, another non-press fit cementless system requires placement of the femoral component along the axis of the femoral neck using a screw fixation implant [45]. Due to the fact that cemented stem position is established by the location and orientation of the femoral osteotomy, it is likely that the findings of the present study will not prove useful for predicting post-operative cemented stem position. The cementless THRs in the present study were performed by a THR surgeon with 14 years of experience consisting of hundreds of cementless THRs. It is plausible that this level of experience led to the subjective identification of trochanter position on pre-operative radiographs, intra-operative adjustment to address trochanter position during femoral canal preparation, and a reduced incidence of malpositioned stems. As such, the effect of trochanter grade on surgical factors and post-operative implant position for a less experienced THR surgeon is unknown. The effect of surgeon experience level on canine cementless stem position should be the focus of future work.

The present study focused on defining a practical trochanter grading system, reporting the incidence of the four trochanter grades in a continuous cohort of cases, and identifying potential interactions with numerous clinical factors. The purpose of this study was not to investigate the effect of trochanter morphology on medium or long-term clinical outcome. The authors considered this decision a priori. Rationale for this decision was to first define a trochanter classification method, report incidence data in a moderately-sized cohort of canine THRs, and screen for potential interactions with other clinical parameters prior to moving to hypothesis-driven studies related to outcome. As such, the effect of trochanter grade on clinical outcome in cementless THR patients remains unknown and may be of interest in future studies.

In view of the large number of statistical tests that were carried out in this study, it is best to regard the outcomes as hypothesis-generating rather than hypothesis-testing analyses. Our results suggest that canine THR patients with Grade IV trochanters are associated with femoral geometrical differences as compared to the other trochanter grades. Moreover, Grade IV cases are more likely to require technique modifications and are associated with prolonged surgical times. The large number of statistical tests and the small number (*n* = 6) of Grade IV cases specifically, implies that our findings should be replicated before being considered secure. Craniocaudal radiographs of the femur were obtained under heavy sedation or anesthesia and used to determine trochanter grade for all cases. It is possible that subtle rotational malpositioning could affect trochanter grade, although radiography has previously been validated to accurately characterize trochanter position in a recent study [39]. Moreover, radiographs were obtained at an academic referral hospital with a high joint replacement caseload by radiology technicians with 10 or more years of experience and with immediate inspection of radiographic positioning by the authors. Lastly, a single investigator (WBS) utilized the grading system to retrospectively score greater trochanter morphology. Radiographs were blinded prior to scoring in an attempt to limit observational bias. Intra- and inter-observer variability was not assessed and should be the focus of future work.

## Conclusion

Classification of greater trochanter morphology in the frontal plane in canine THR patients may prove useful during pre-operative THR templating and surgical planning. While Grade IV trochanters were uncommon in this cohort of THR patients (6/150 THRs, 4%), these cases were associated with prolonged surgical times and technique modifications during femoral broaching. Trochanter grade was associated with differences in post-operative cementless stem position.

## Methods

### Inclusion and exclusion criteria

Medical records from a university veterinary teaching hospital from 2013 to 2020 were reviewed. This timeframe was selected in as it included a continuous cohort of cementless THRs performed by a single surgeon using a commercially available THR system (BFX™ Universal THR, Biomedtrix, Whippany, NJ). Additional criteria included availability of complete sets of pre-operative and post-operative THR radiographs [4, 18], anesthetic record, and operative report. Dogs receiving cemented (CFX™) stems or cemented Micro/Nano™ stems were not included. If an individual had surgery on both hips, each joint was considered separately.

### Data collection

Demographic data collected included age, sex, breed, and body weight (kg) at the time of THR. The underlying diagnosis leading to THR was also categorized as one of the following: hip dysplasia with secondary osteoarthritis, hip dysplasia with the luxoid phenotype [46], traumatic hip luxation, or fracture of the femoral head, neck, or acetabulum. Surgical time, stem size, stem type (BFX™-standard, BFX™-collared, BFX™-lateral bolt), technique modifications during femoral preparation (e.g. use of aggressive-toothed canal files, Hall® pneumatic high-speed burr, or GTO) (Supplemental Figure 1), and intra-operative complications were recorded.

### Pre-operative classification of greater trochanter morphology

**Fig. 1:**
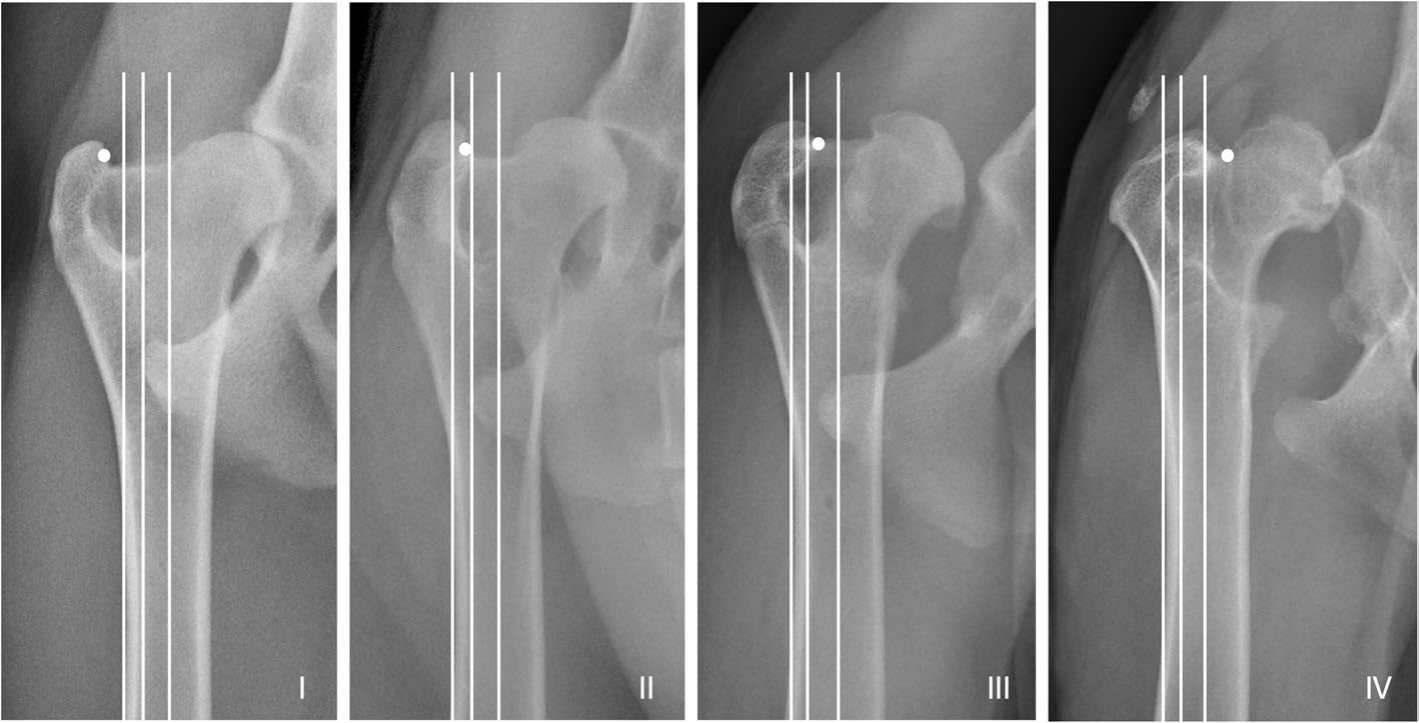
Greater trochanter classification system adapted from human THR. On the craniocaudal radiograph (frontal plane), the anatomic axis of the femur was first determined. Two additional axes were created parallel to the anatomic axis. The first of these axes was placed tangential to the periosteal surface of the lateral cortex at the femoral isthmus, while the second was placed tangential to the endosteal surface of the lateral cortex at the femoral isthmus. These three parallel axes created four zones for trochanter classification (Grade I-IV) from the lateral to medial direction. The medial-most aspect of the greater trochanter was used as the reference point for classification

One author (WBS) evaluated all radiographs retrospectively and was blinded to patient identification. Radiographs were calibrated using a 10 cm calibration marker (Biomedtrix) and measured using eFilm Workstation 3.3 (VCA Antech, Los Angeles, CA). Using the pre-operative craniocaudal view, trochanter morphology was classified in the frontal plane using an ordinal system adapted from human THR studies (Fig. 1) [29]. The anatomic axis of the femur was first determined as previously described [47]. Two additional axes were created parallel to the anatomic axis. One of these axes was placed tangential to the periosteal surface of the lateral cortex at the femoral isthmus, while the other was placed tangential to the endosteal surface of the lateral cortex at the femoral isthmus. These three parallel axes created four zones for trochanter classification (Grade I-IV) from the lateral to medial direction. The medial-most aspect of the greater trochanter was used as the reference point for grading (Fig. 1).

### Additional pre-operative and post-operative radiographic assessment

Additional pre-operative radiographic measurements were performed to screen for potential associations between trochanter grade and femoral geometry. Distal femoral varus was assessed using the anatomic lateral distal femoral angle (aLDFA) as previously described [47]. Angle of inclination was determined as previously described [48]. Canal flare index (CFI) and cortical thickness ratio (CTR) were determined as previously described [20, 49, 50]. Femoral torsion angle (FTA), which is a measurement of femoral head and neck anteversion, was calculated from craniocaudal and mediolateral radiographs using the trigonometric method [41, 42].

Data collected from post-operative radiographs included canal fill, depth of stem insertion relative to the proximal greater trochanter, stem alignment, deviation of the central axis of the stem from midline, and stem version [8, 14, 20]. For stem alignment, a positive value denoted a varus position and a negative value denoted a valgus position in the frontal plane (craniocaudal view). In the sagittal plane (mediolateral view), a positive value denoted a stem placed in a cranioproximal to caudodistal direction, while a negative value denoted a stem placed in a caudoproximal to craniodistal direction. For stem translation, a positive value denoted a medially translated stem and a negative value denoted a laterally translated stem (craniocaudal view). A positive value denoted a cranially translated stem and a negative value denoted a caudally translated stem (mediolateral view). Stem anteversion was determined relative to the anatomic axis of the femur and not the long axis of the cementless stem [42].

### Statistical analysis

All datasets were examined for normality using tabulation, visual examination of histograms and, where necessary, the D’Angostino and Pearson test. When possible, non-normally distributed data were transformed (e.g. log transformation, inverse); alternatively, non-parametric methods of analysis were used. Normally distributed data were reported as mean and standard deviation, non-normally distributed data were reported as median and interquartile range. The associations between various demographics, surgical factors, and pre- or post-operative measurements with Grade I-IV trochanter morphology were examined using analysis of variance (for normal data) applying Scheffe’s post hoc test where appropriate, non-parametric equivalents (for non-normal data) or chi-squared (for ordinal data). Stata 14 (StataCorp, College Station, TX) was used for descriptive and analytical statistics. Significance was assumed at *P* ≤ .05.

## Supplementary Information

**Additional file 1 **: **Supplemental Table 1.**Complete breed list for 135 dogs undergoing 150 cementless total hip replacements.

**Additional file 2 **: **Supplemental Figure 1.** Technique modifications during femoral canal preparation for cementless total hip replacement.

## Data Availability

The datasets generated and/or analyzed during the current study are available from the corresponding author on reasonable request.

## Abbreviations

aLDFA: Anatomic lateral distal femoral angle
BFX: Biologic fixation
CT: Computed tomography
CFI: Canal flare index
CTR: Cortical thickness ratio
FTA: Femoral torsion angle
GTO: Greater trochanter osteotomy
THR: Total hip replacement
Ti-EBM BFX: Titanium electron beam melted BFX
