# Supplementary material for: Greater trochanter morphology and association with patient demographics, surgical factors, and post-operative stem position: a retrospective assessment of 150 cementless THRs in 135 dogs
Source: BMC Vet Res. 2022 Feb 23;18:78. doi: 10.1186/s12917-022-03174-y (PMC8864880; doi:10.1186/s12917-022-03174-y)
Supplement: Supplementary file 1 — Additional file 1 : Supplemental Table 1.Complete breed list for 135 dogs undergoing 150 cementless total hip replacements. [file 12917_2022_3174_MOESM1_ESM.docx]

**Supplemental Table 1:** Complete breed list for 135 dogs undergoing 150 cementless total hip replacements.

| Breed | Number |
| --- | --- |
| Labrador Retriever | 28 |
| German Shepherd | 14 |
| Golden Retriever | 11 |
| Mixed Breed Dog | 8 |
| Pit Bull Terrier | 7 |
| Great Pyrenees | 6 |
| Australian Shepherd | 5 |
| Border Collie | 5 |
| Rottweiler | 5 |
| Australian Cattle Dog | 4 |
| Cane Corso Mastiff | 4 |
| Husky | 3 |
| Saint Bernard | 3 |
| Bernese Mountain Dog | 2 |
| Black Mouth Curr | 2 |
| Bouvier Des Flanders | 2 |
| Catahoula | 2 |
| Coonhound | 2 |
| German Shorthaired Pointer | 2 |
| Great Dane | 2 |
| Norwegian Elkhound | 2 |
| Alaskan Malamute | 1 |
| Anatolian Shepherd | 1 |
| Belgian Malinois | 1 |
| Bloodhound | 1 |
| Boxer | 1 |
| Boykin Spaniel | 1 |
| Goldendoodle | 1 |
| Labradoodle | 1 |
| Leonberger | 1 |
| Newfoundland | 1 |
| Poodle | 1 |
| Samoyed | 1 |
| Schnauzer | 1 |
| Shiba Inu | 1 |
| Turkish Boz Shepherd | 1 |
| Weimaraner | 1 |
| Total | 135 |
