# Supplementary figures and images for: Greater trochanter morphology and association with patient demographics, surgical factors, and post-operative stem position: a retrospective assessment of 150 cementless THRs in 135 dogs
Source: BMC Vet Res. 2022 Feb 23;18:78. doi: 10.1186/s12917-022-03174-y (PMC8864880; doi:10.1186/s12917-022-03174-y)

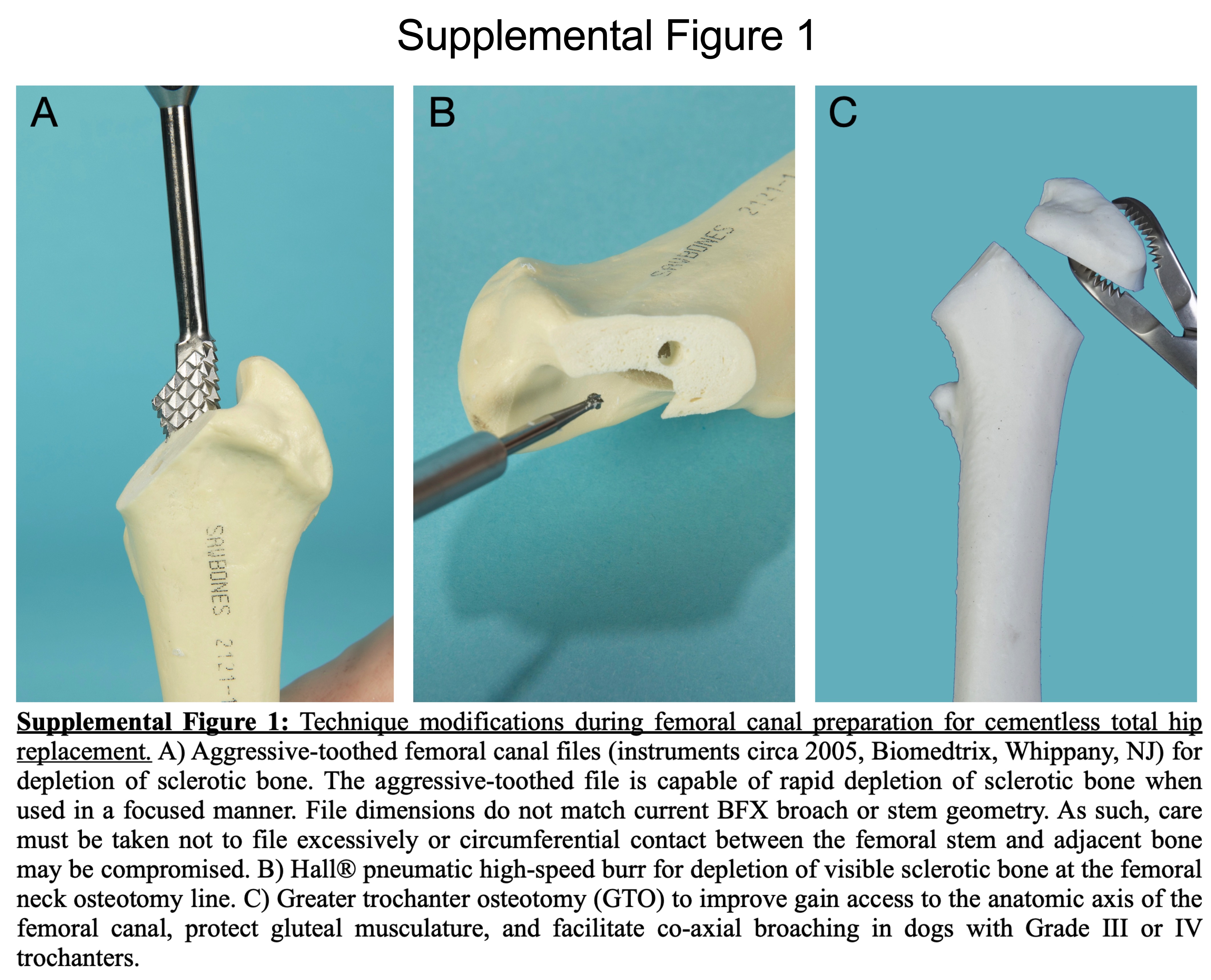

Supplement: Supplementary file 2 — Additional file 2 : Supplemental Figure 1. Technique modifications during femoral canal preparation for cementless total hip replacement. [file 12917_2022_3174_MOESM2_ESM.jpg]
